# Comparing urine samples and cervical swabs for Chlamydia testing in a female population by means of Strand Displacement Assay (SDA)

**DOI:** 10.1186/1472-6874-10-9

**Published:** 2010-03-25

**Authors:** Siren Haugland, Turid Thune, Beata Fosse, Tore Wentzel-Larsen, Stig Ove Hjelmevoll, Helge Myrmel

**Affiliations:** 1 Department of Public Health, University of Bergen, Norway; 2 Department of Dermatology and Venereology, Haukeland University Hospital, Bergen, Norway; 3 Department of Microbiology and Immunology, Haukeland University Hospital, Bergen, Norway; 4 Centre for Clinical Research, Haukeland University Hospital, Bergen, Norway; 5 Department of Microbiology and Infection Control, University Hospital of North Norway, Tromsoe, Norway

## Abstract

**Background:**

There has been an increasing number of diagnosed cases of *Chlamydia trachomatis *in many countries, in particular among young people. The present study was based on a growing request to examine urine as a supplementary or primary specimen in screening for *Chlamydia trachomatis *in women, with the Becton Dickinson ProbeTec (BDPT) Strand Displacement Assay (SDA). Urine samples may be particularly important in screening young people who are asymptomatic.

**Methods:**

A total of 603 women aged 15 and older were enrolled from the Sexually Transmitted Infection (STI) clinic at Haukeland University Hospital, Norway, in 2007. Only 31 women were older than 35 years. Cervical swabs and urine samples were tested with BDPT for all participants. In cases of discrepant test results from a given patient, both samples were retested by Cobas TaqManCT and a Polymerase Chain Reaction (PCR)-method (in-house). Prevalence of *C. trachomatis*, sensitivity, and specificity were estimated by latent class analysis using all test results available. Bootstrap BC confidence intervals (10 000 computations) were estimated for sensitivity and specificity, and their differences in cervix vs. urine tests.

**Results:**

A total of 1809 specimens were collected from 603 patients. 80 women (13.4%) were positive for *C. trachomatis*. Among these, BDPT identified 72 and 73 as positive in cervix and urine samples, respectively. Of the 523 *C. trachomatis *negative women, BDPT identified 519 as negative based on cervical swabs, and 514 based on urine samples. Sensitivity for cervical swabs and urine samples with the BDPT were 89.0% (95% CI 78.8, 98.6) and 90.2% (95% CI 78.1, 95.5), respectively. The corresponding values for specificity were 99.2% (95% CI 98.3, 100) and 98.3% (95% CI 96.4, 100).

**Conclusions:**

This study indicates that urine specimens are adequate for screening high-risk groups for *C. trachomatis *by the SDA method (BDPT). Such an approach may facilitate early detection and treatment of the target groups for screening, and be cost-effective for patients and the health services.

## Background

An increased number of diagnosed cases of *C. trachomatis *in younger age groups calls for an intensified effort to prevent infections, and complications such as pelvic inflammatory disease and infertility. In 2007, a total of 22 847 infections were reported in Norway, constituting 8.4% of the individuals tested. Of those infected, 61% were women [1]. Of the total number of reported infections, 68% were younger than 25 years (40% were between 20-24 years, and 28% between 15-19 years). The greatest increase in the number of infections was seen among 15-19 year-olds. Similar trends are reported from Sweden, Denmark, England and the US [2-7].

Most *C. trachomatis *infections are asymptomatic. Thus, motivation for testing is based on the patients' knowledge of transmission and consequences of infection, as well as perceived barriers. Screening of asymptomatic populations is facilitated by the use of minimally invasive or non-invasive sampling. Most individuals tested for *C. trachomatis *in Norway are above 24 years, but the prevalence rates are significantly higher between 15 and 24 years [1]. Thus, it is important to offer sampling procedures which lower the threshold for screening and diagnostic testing in the younger age groups as well as being cost-effective. Gynaecological examination is generally considered a main barrier in recruiting young women for testing [8].

Endocervical swabs, vaginal swabs and urine are potential specimens for testing women. The diagnostic value of each of these specimens may vary according to the population tested and laboratory method used. Several laboratory methods are available for direct detection of *C. trachomatis*, such as cell culture, immunofluorescense microscopy and enzyme immunoassays (EIA). At present, nucleic acid amplification tests (NAATs) provide unsurpassed sensitivity and high specificity when applied on cervical and urethral swabs and male urine samples. NAATs target species-specific sequences of DNA for detection of pathogens. For the commonly used polymerase chain reaction (PCR) method, there is evidence that testing of female and male urine is equivalent to cervical and urethral swabs, respectively, in terms of sensitivity and specificity [9-11]. Vaginal swabs are not validated for use by the manufacturers of the two most commonly used NAATs in Norway. The BDProbeTec™ Chlamydia trachomatis Amplified DNA Assay (BDPT) utilizes a strand displacement amplification (SDA) method coupled with a fluorescent energy transfer (ET) measurement in detecting the amplified product. For this method as well, the sensitivity in male urine samples is comparable to that of urethral swabs. However, studies based on urine samples from women have shown lower sensitivity compared to endocervical swabs [12,13], a potential impediment for testing, especially for screening purposes.

The aim of the present study was to evaluate the performance of the SDA method in detection of *C. trachomatis *in cervical swabs and urine samples in a female population. This was achieved by comparing discrepant results in the BD SDA to a composite reference standard defined by results from two other NAATs, the Cobas TaqMan CT PCR and an in-house PCR targeting genomic *C. trachomatis *DNA.

## Methods

### Ethics

The study was approved by the regional ethics committee and the involved institutions. The women were included on the basis of verbal consent, after receiving oral and written information about the study, and their right to receive standard testing and treatment without taking part in the study. Patient information was collected and stored at the hospital. All participants received the standard testing, and in addition an extra cervical swab and a urine sample were collected at the time of consultation. Participation was not expected to burden the women.

### Study population

Women attending the STI clinic at Haukeland University Hospital, Bergen, Norway, were enrolled in the study. This clinic has a drop-in policy, providing testing of anyone worried about *C. trachomatis *or other sexually transmitted infections (STI), without an appointment or referral during daytime opening hours. Both examination and treatment are free of charge for the patient. A total of about 2000 women are tested for *C. trachomatis *in this clinic every year. Among these, 13% are positive for *C. trachomatis*.

### Participants

All women (n = 618) tested for *C. trachomatis *at the STI clinic in the period from January 2nd to June 15th, 2007, were asked to participate. Reasons for contacting the clinic were symptoms, contact tracing or unprotected sex. A medical doctor evaluated the need for *C. trachomatis *testing. Exclusion criteria were pregnancy or the use of antibiotics during the previous 21 days.

### Specimen collection

Participants received standard clinical examination, testing and treatment. Two cervical swabs were collected during the examination, and the patients also delivered a urine sample. Vaginal swabs were not collected since BDPT is not approved for this specimen.

Urine samples were collected according to guidelines (first catch urine: FCU, mid-stream, at least two hours after previous urination, at least 10 - 20 ml urine). Cervical swabs were collected by the doctors at the clinic, based on regular procedures for speculum examination and using the manufacturer's collection kits. After cleaning of the cervix with the dry swab provided in the kit, two swabs were collected for each patient. One swab was for the BDPT medium, and one for Roche CT. The two swabs were collected first and second alternately, to minimize the effect of sample variation. The specimens were transported to the diagnostic laboratory at the microbiology department on the same campus. The urine specimens were transported without any added transport medium, and refrigerated and examined within 48 hours with BDPT, and within 7 days with Cobas TaqMan CT and in-house PCR. The cervical swabs were stored at room temperature and tested within 48 hours with BDPT and within 10 days with Cobas TaqMan CT and in-house PCR. When necessary, additional tests for other STIs were collected after the tests above. Patients were tested for *Trichomonas vaginalis, Herpes simplex 1 *and *2, Neisseria gonorrhoea, HIV, Hepatitis B and C*, and *Treponema pallidum *when indicated.

### Testing of specimens

Cervical swabs and urine samples were tested with BDPT for all participants, according to the manufacturers' guidelines and standard routines at the laboratory. Internal validation was carried out by adding an amplifying control (AC) for all the examined tests.

In cases of discrepant test results for the cervical swab and urine samples from a given patient, both samples were retested by Cobas TaqManCT in the same laboratory, and also tested by a PCR-method (in-house) developed at the University Hospital of North Norway.

A woman was considered *C. trachomatis *positive if both FCU and the cervical swab were positive by the SDA method (BDPT), or if one of the specimens tested positive by the SDA method and at least one specimen was positive for the Cobas TaqManCT or the in-house PCR.

### Laboratory methods

The BD ProbeTec™ (Chlamydia trachomatis amplified DNA assay, Becton, Dickinson and Company, Sparks, MD USA) utilizes homogeneous Strand Displacement Amplification (SDA) technology as the amplification method and fluorescent energy transfer (ET) as the detection method to test for the presence of *C. trachomatis*. The test is based on the simultaneous amplification and detection of target DNA, using amplification primers directed against plasmid DNA and a fluorescent labelled detector probe. The COBAS TaqMan CT Test (Roche Diagnostics GmbH, Mannheim, Germany) is based on manual specimen preparation to obtain *C. trachomatis *DNA, followed by PCR amplification of target DNA. The PCR uses *C. trachomatis*-specific complementary primers directed against cryptic plasmid DNA. The result is obtained by detection of a cleaved dual fluorescent dye-labelled oligonucleotide detection probe that permits the detection of *C. trachomatis *target amplified product (amplicon) and *C. trachomatis *Internal Control DNA, which is amplified and detected simultaneously with the specimen. Both methods were performed according to the manufacturer's instructions. Both BDPT and the in-house PCR were able to detect the new variant of *C. trachomatis*.

### Statistical analysis

Descriptive statistics included frequency distributions. Prevalence of *C. trachomatis *and sensitivity and specificity of the BDPT cervix and urine samples were estimated by latent class analysis using all test results available, independent of the above classification of discrepant results [14]. Bootstrap BC_a _confidence intervals [15], a procedure that does not make specific distributional assumptions, were computed for sensitivity and specificity, and their differences in cervical vs urine tests. A total of 10 000 bootstrap computations were undertaken.

All tests were two-sided, and p-values below 0.05 were considered significant. Descriptive analyses used SPSS version 15.0 (SPSS Inc, Chicago, IL, USA). Latent class analysis used the package poLCA[15] and bootstrap BC_a _intervals were computed in the package boot in R (The R Foundation for Statistical Computing, Vienna, Austria).

## Results

A total of 1809 specimens were collected from 603 patients, all of which were included in the statistical data analyses. Among the participants, 80 women (13%) were 15-19 years old, 235 (39%) were 20-22 years, 154 (26%) were 23-25 years, 63 (10%) were 26-29 years, 39 (7%) were 30-34 years. The remaining 32 women were between 35 and 56 years. A total of 15 women were excluded; ten due to lack of an adequate urine sample, three due to use of antibiotics, and two because of inadequate gynaecological examination (one woman had painful genital herpes, one did not want an examination). None of the attending women were excluded due to pregnancy.

No inhibitory effect was observed in the samples in any of the methods. Based on the definition of a positive test (*C. trachomatis *positive in BDPT in both urine and cervical swab or one positive test in any two specimens in two out of three methods applied), 80 women (13.4%) were defined as positive for *C. trachomatis*, and 523 were negative. Of the 80 *C. trachomatis *positives, BDPT identified 72 and 73 as positive in cervix and urine samples, respectively. Of the 523 *C. trachomatis *negative women, BDPT identified 519 as negative based on cervical swabs, and 514 based on urine samples.

Three of the *C. trachomatis *positive women were diagnosed with genital condyloma acuminata. None were diagnosed with other STI. Of the *C. trachomatis *negative women 35 had condyloma acuminata, seven genital herpes and one trichomonas vaginalis. Two were hepatitis C positive.

Table 1 shows the results of the SDA method according to specimen. BDPT gave discrepant results for 28 patients, and their samples were subsequently tested with the two PCR methods.

**Table 1:** Results of testing for Chlamydia trachomatis by BD ProbeTecET according to specimen for a total of 603 women

	Number of women, N (%)
Cervix neg, urine neg	510 (84.6)
Cervix neg, urine pos	17 (2.8)
Cervix pos, urine neg	11 (1.8)
Cervix pos, urine pos	65 (10.8)

Total	603 (100)

Table 2 shows the results of the additional tests and the conclusion (presence or absence of *C. trachomatis *infection) for these 28 women. For 15 *C. trachomatis *positive women, *C. trachomatis *was detected in either FCU (seven women) or the cervical swab (eight women) by the SDA method.

**Table 2:** Results of the BD Probe Tec ET, Cobas Taq Man and in-house PCR for 28 women tested by all three methods

Number of same inter-method pattern	BD Probe tec		Cobas TM		In-house PCR		Overall result
	**urine**	**cervix**	**urine**	**cervix**	**urine**	**cervix**	

1	**-**	**+**	**+**	**+**	**-**	**-**	**+**
4	**-**	**+**	**-**	**-**	**-**	**-**	**-**
4	**-**	**+**	**+**	**+**	**+**	**+**	**+**
1	**-**	**+**	**-**	**+**	**-**	**+**	**+**
1	**-**	**+**	**-**	**+**	**+**	**-**	**+**
1	**+**	**-**	**+**	**-**	**+**	**-**	**+**
2	**+**	**-**	**+**	**+**	**+**	**-**	**+**
1	**+**	**-**	**+**	**+**	**-**	**+**	**+**
9	**+**	**-**	**-**	**-**	**-**	**-**	**-**
4	**+**	**-**	**+**	**-**	**+**	**-**	**+**

Based on the latent class analyses the estimated prevalence of *C. trachomatis *was 13.4%. There were no significant differences in diagnostic outcome between cervical swabs and urine samples using the SDA technique. Estimates including confidence intervals are summarised in Table 3.

**Table 3:** Results of sensitivity and specificity analyses for the SDA method, including 95% Confidence Intervals (CI)

	% sensitivity	95% CI	% specificity	95% CI
Cervix	89.0	(78.8, 98.6)	99.2	(98.3, 100)
Urine	90.2	(78.1, 95.5)	98.3	(96.4, 100)
difference (C-U)	-1.2	(-12.3, 16.2)	1.0	(-1.3, 3.3)

Positive and negative predictive values (PPV and NPV) were estimated based on prevalence, sensitivity, and specificity. For cervical swabs the PPV and NPV were 94.7% and 99.2%, respectively. The corresponding values for urine samples were 89.0% and 98.3%

## Discussion

This study shows that the SDA method has comparable sensitivity and specificity in cervical swabs and urine specimens for *C. trachomatis *testing. This suggests that urine is an acceptable alternative to cervical swabs in the studied female sample. The confidence interval for difference in sensitivity was broad, and differences in sensitivity in cervix and urine samples cannot be ruled out. On the other hand, substantial differences in specificity between the two tests are unlikely. Both the present and previous studies [16] show that some infected individuals are positive only with FCU. To maximize diagnostic sensitivity, both urine and cervical swabs could be sampled when possible.

The results are based on testing of women in a university hospital STI clinic and laboratory, with well-established procedures for testing and handling specimens. The sampling was carried out by specialist medical doctors. The limitations of this study are mainly related to the potential bias of a discrepant analysis approach. Additional tests with COBAS TaqMan CT Test and the in-house PCR were carried out for discrepant tests only. The aim of the present study was to evaluate the performance of the SDA method in detection of *C. trachomatis *in cervical swabs and urine samples, not to compare or evaluate the three methods. The differences in sensitivity and specificity are expected to be small when the frequency of discordant results is relatively low [14]. Three sets of tests for all patients would have given a stronger design resulting in higher precision in our estimates, as there is a possibility of a *C. trachomatis *diagnosis, even if both urine and the cervical swabs are negative in the SDA test. A possible bias in our latent class etimates might occur if the additional Cobas TaqMan CT and PCR tests are systematically different when the BDPT tests are concordant, respectively discordant. We do not expect that such systematic differences are likely.

Previous studies have shown a higher sensitivity of the SDA method for cervical swabs than for FCU [12,13]. Our study suggests that urine may be used for screening, as there was no significant difference between the two specimens. From a clinical perspective, the positive predictive value (PPV) of a test determines the proportion of patients with positive test results who are correctly diagnosed as having the infection. In the present study 94.7% were diagnosed correctly by cervical swabs and 89.0% based on FCU. Thus, both urine and cervical samples are useful for diagnostic purposes.

The proportion of positive tests in the studied population is expected to be higher than in the general population, as the sample is recruited from a STI clinic. However, several studies suggest that the target populations for screening, such as young people and individuals with multiple or frequent change of sexual partners, have comparable or higher prevalence rates [17,18]. In populations with lower prevalence rates, i.e. pregnant women with *C. trachomatis *infection rates of 3-5%, PPV would be significantly lower. Thus, a greater proportion of women would receive false positive tests than in high prevalence groups [19]. On this basis, confirmation of a positive test, by sampling both urine and cervical swabs and retesting of positive tests, should be considered in screening low-prevalence groups.

Early detection and treatment of *C. trachomatis *may reduce complications resulting from untreated infections. From a public health point of view, current recommendations emphasise that high risk groups should be approached and offered non-invasive testing. The present findings are important to facilitate such intervention. Studies of cost-effectiveness also support examination of FCU in screening high-risk populations [20].

Previous studies have argued for self obtained vaginal swabs in screening for *C. trachomatis *in female populations [21-23]. Blake demonstrated higher sensitivity and specificity for self obtained vaginal swabs when compared to first cast urine and endocervical samples [21]. Schachter and colleagues found that vaginal swabs identified as many infected patients as cervical swabs, and more than urine samples. These studies have also demonstrated cost-effectiveness. Michel (2007) recommended vaginal swabs based on the findings that FCU had lower organism load than vaginal swabs in asymptomatic women [22]. However, vaginal swabs are not validated by the manufacturers of the two most commonly used NAATs in Norway.

For boys, FCU is the sample of choice. In advocating screening of asymptomatic young people, there may be advantages in promoting the same methods of sampling both for boys and girls. Earlier research showed that testing strategies targeting asymptomatic individuals in established community and clinical settings, are more likely to yield high acceptance rates than home based approaches [24]. Testing of FCU may allow for express visit options for clients with low risk of other STIs, increasing the efficiency of outpatient clinics [24-26].

In promoting screening among high-risk groups, it is important that information is linked to balanced information about other sexually transmitted infections. Fear of the consequences of *C. trachomatis *may promote condom use. Thus, it is important to avoid a decrease in use of condoms due to a perception of easy testing and treatment for *C. trachomatis*.

## Conclusions

This study suggests that urine specimens are comparable to cervical swabs in screening high-risk groups for *C. trachomatis *by the SDA method (BDPT). Such an approach may facilitate early detection and treatment of the target groups for screening, and be cost-effective for patients and the health services. Studies with additional tests performed for a larger proportion of the patients, may reduce the uncertainty related to differences in test sensitivity. More research is needed to determine the usefulness of urine samples in testing low-prevalence populations.

## Competing interests

The authors declare that they have no competing interests.

## Authors' contributions

SH, TT, BF, and HM contributed to the design of the study. All authors contributed to the writing process. HM and BF carried out the laboratory analyses at the Haukeland University Hospital. SOH conducted the in-house PCR analyses. TWL and SH carried out statistical analysis, TWL performed the Bootstrap analysis. All authors read and approved the final manuscript.

## Pre-publication history

The pre-publication history for this paper can be accessed here:

http://www.biomedcentral.com/1472-6874/10/9/prepub
